# An Experimental Investigation on Micro End Milling with High-Speed Up Cut Milling for Hardened Die Steel

**DOI:** 10.3390/ma13214745

**Published:** 2020-10-23

**Authors:** Haruki Kino, Takumi Imada, Keiji Ogawa, Heisaburo Nakagawa, Hitomi Kojima

**Affiliations:** 1 MOLDINO Tool Engineering Ltd., Hulic Ryogoku Building 8F, 4-31-11, Ryogoku, Sumida-ku, Tokyo 130-0026, Japan; hkino@moldino.com; 2 Industrial Research Center of Shiga Prefecture, 232 Kamitoyama, Ritto, Shiga 520-3004, Japan; imada.takumi@shiga-irc.go.jp; 3 Ryukoku University, 1-5 Yokotani, Seta Oe-cho, Otsu, Shiga 520-2194, Japan; 4 Nakagawa Machining R&D Center, 974-1 Nakanoshima, Wakayama 640-8392, Japan; nakagawa@mech.usp.ac.jp; 5 Big Daishowa Seiki Co Ltd., 3-3-39 Nishi-ishikiri-cho, Higashi-Osaka, Osaka 579-8025, Japan; moda0801@yahoo.co.jp

**Keywords:** end mill, high-speed cutting, high-feed cutting, cutting force, surface roughness, tool wear, tool life, machining accuracy, precise machining

## Abstract

The importance of micromachining using small diameter end mills and the dies used for them has been increasing in the machining of small parts. However, the reality is that there are various requirements to improve the machining surface, machining accuracy, machining efficiency, and tool life. Therefore, this paper discusses the possibility of satisfying these requirements by high-speed up cut milling in side cutting. The goal of this study was to solve the aforementioned problems, by conducting a detailed analysis of the machining phenomena in order to understand their mechanisms. In particular, the effects of high-speed cutting using a high-speed air-turbine spindle with highly stiff rolling bearings were analyzed. Moreover, cutting experiments were conducted by measuring the cutting force and flank wear of the tool, to reveal the differences in the cutting phenomena relative to the cutting direction in high-speed micro end milling. Description of the machined surface and the measurement of its profile were also included in the discussions. On the basis of the results, high-speed up cut milling is a better choice than down cut milling; furthermore, a high-feed rate further increases machining efficiency and improves tool life.

## 1. Introduction

In recent years, there has been a continuous drive toward miniaturization and high-precision platforms for medical; optical; and electronic devices, parts, and equipment. To meet the demand for shortening the delivery time of dies required for the manufacture of these products and parts, not only high accuracy but also high-efficiency machining is imperative. With this, it is desirable to establish an effective machining method such as an advanced micro end milling technology. The most commonly used method in end milling on high-speed machining centers is down cut milling. However, in the past, up cut milling was sometimes used because of the backlash of the machine and the characteristics of the cutting tool material. It is only in recent years that down cut milling has been recognized as the normal machining method, owing to its longer tool life and better machining accuracy. In the case of precision machining of a vertical wall profile, it is often more practical to apply contour machining to adjust to the requirement of high machining accuracy and good surface roughness for the vertical wall. For a micro end mill, the contour and down cut methods are generally used in consideration of the stiffness of the tool; however, these could be accompanied by some issues in machining efficiency and the machined products, which would demand technical improvements. This suggests the implied difficulty in machining with high accuracy due to the small diameter of the cutting tool, which has low stiffness and deforms easily during the process. In addition, preventing the loss of accuracy due to deformation and avoiding tool damage are normally addressed by setting the depth of cut to a small value, which makes highly efficient machining difficult to perform at present. These shortcomings were a major consideration in the active research and development of micro end milling. Some of these papers explored the aspects of machining quality, i.e., surface properties and machining accuracy, and machining phenomena, i.e., cutting force and tool wear. For example, W. Chen et al. [1] tried to achieve surface generation modeling for micro end milling, and S.P. Leo [2] considered an empirical model for the optimization of surface roughness and machining time parameters using genetic algorithm. T. M. Moges et al. [3] performed an analytical investigation on machining accuracy, considering cutter runout and elastic recovery in micro end milling, and D. Goto et al. [4] focused on tool runout for their experimental studies on machining accuracy. Moreover, some papers have proposed the use of analytical cutting force models [5,6,7,8]; prediction of these cutting forces during micro end milling considering chip thickness [9,10]; and the modeling of the effects of runout, minimum chip thickness, and elastic recovery on the cutting forces [11,12,13,14]. However, these models were only able to explain the initial condition of the cutting tool, whereas the actual machining phenomena changes with the tool wear continued to progress. M. Bohley et al. [15] and C. Bandapalli et al. [16] conducted a study on the conditions around tool wear during the micro end milling of a Ti-alloy, and I. G. Reichenbach [17] showed tool life criteria and the wear behavior of single-edge ultra-small micro end mill for polymethyl methacrylate. Moreover, K. Vipindas [18] and L. Alhadeff [19] investigated changes in machining performance considering tool wear in a Ti-alloy and brass, respectively, and N. Swain [20] experimentally studied the machining characteristics of a Ni-alloy using micro end mills. Nevertheless, there are only a few papers to date that have dealt with the actual machining phenomena of a hardened die steel, a material widely used for the manufacture of industrial parts. Previously [21], it was reported in many papers that report on the tool wear, deterioration of machined surface roughness due to the increase of cutting force, and the deterioration of machined surface quality due to the progress of tool wear in the machining of large diameter end mills such as 2 mm in diameter or more. G. E. D’Errico et al. [22] studied tool wear during end milling [22,23,24,25,26]. D. A. Axinte et al. [27] investigated machining qualities such as surface integrity after end milling [27,28,29,30]. D. A. Axinte et al. [27] studied the effect of cutting length and feed rate on machined surface roughness but did not mention the relationship with tool wear. M. C. Kang et al. [28] mainly evaluated the change in machined surface roughness with cutting length depending on the coating material type on the end mill but did not reach an evaluation on machining accuracy. L. Ning et al. [29] and I. Buj-Corral [30] modeled tool wear and machined surface properties respectively are compared with experimental results, but the conditions were limited. However, the conventional cutting theory and evaluation methods are not applicable to fine and precise machining because the cutting fields of a large diameter tool such as 2 mm or more is quite different from that of a small diameter end mill. For example, in the case of small diameter end milling, the effect of the tool wear and definition of life of the tool is different because the depth of cut is extremely small. Furthermore, in order to evaluate the cutting performance of a small diameter end mill, it is necessary to increase the cutting speed by using a high-speed spindle. The diameter of the end mill used in this study is 0.5 mm, and the cutting phenomena with such a small diameter end mill with a large axial depth of cut of 0.5 mm (= 1*D*), which is equal to the tool diameter *D*, had not been studied in the past. In this study, a cutting speed range of 157 m/min, which is considered to be good for conventional cutting in practical terms, was achieved in small diameter end milling, but the phenomena of machining by high-efficiency up cut milling had not yet been clarified. The present study aims to achieve the requirements for high-efficiency and high-quality in the field of micro-precise machining, and to clarify the phenomena that occur during machining by detailed verification. Therefore, the present authors conducted a study on the micro end milling of a 0.5 mm diameter hardened die steel during side cutting; measured the actual tool profile and wear in detail; and examined the changes in the cutting force, the amount of residual stock removal, and the machined surface roughness as the tool wear progressed [21]. The focus herein revolves around the effects of high-speed cutting in micro end milling, of the application of a high-speed spindle with rolling bearings. The cutting experiments include measuring the cutting force and flank wear of the tool, which is essential in determining the differences in cutting phenomena relative to the cutting direction, during the high-speed micro end milling. The influence of feed rate is also evaluated.

## 2. Experimental Methods

**Figure 1:**
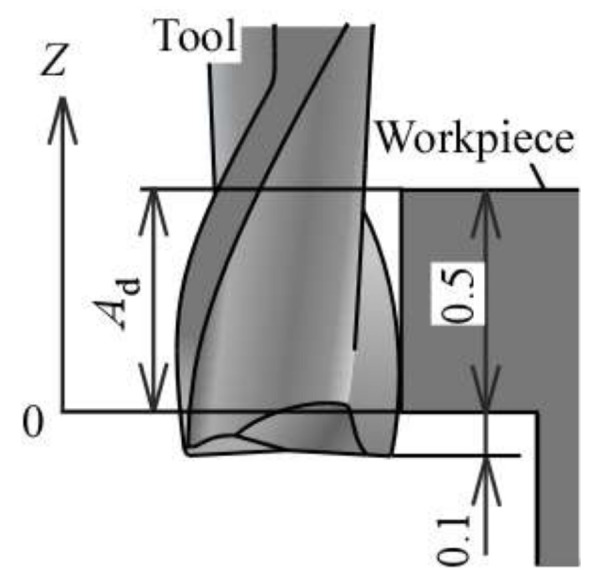
Interference situation between the workpiece and the tool.

**Figure 2:**
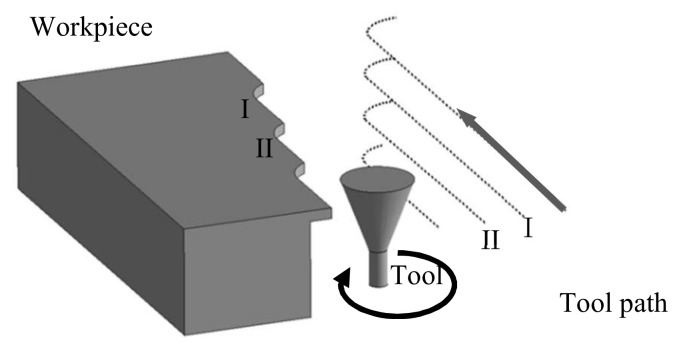
Cutter location for experiments in the case of up cut milling.

A 0.5 mm diameter square end mill made of TiSiN-coated cemented carbide was used as a micro end mill tool. There were two flutes in this tool. A high-precision machining center YMC325 (Yasuda Precision Tools K.K., Okayama, Japan), with a maximum spindle speed of 30,000 min^−1^, was used for the cutting tests. The cutting force was measured while a workpiece was cut on a high-precision dynamometer 9265C1 (Kistler, Winterthur, Switzerland). The workpiece material was a hardened die steel and a well-known die material (JIS: SKD61) of HRC53 hardness. The interference situation between the workpiece and the tool is described in Figure 1. To simplify the analysis of the cutting phenomena, the side cutting was performed using only the outer peripheral flutes with a 0.1 mm protruding edge. Figure 2 illustrates the cutter location for the experiments in the up cut milling, under the following specific procedure. First, a workpiece was surfaced for use in making measurements for a tool in new condition. Then, the workpiece for producing tool cutting edge wear was cut for a prescribed distance. After that, the workpiece was again cut to fabricate a post-wear reference surface I. Next, the post-wear reference surface I was machined repeatedly until steady-state cutting resulted, thus obtaining the steady-state cutting surface II. Next, the normal and tangential components of cutting force, $F_{n}$ and $F_{t}$, were monitored during steady cutting. Then, the normal and tangential components of was defined as the average cutting force, $\bar{F_{n}}$ and $\bar{F_{t}}$, and evaluated. Finally, the surface roughness in the feed direction of the tool was measured on the steady cutting surface using a surface texture measuring instrument SV3100C (Mitutoyo Corp., Kanagawa, Japan). The list of the cutting conditions is given in Table 1. First, the cutting length at which a steady cutting surface is obtained, i.e., the cutting phenomenon at the initial stage of cutting, was evaluated. The cutting length of 0 m means this initial stage of cutting. Up and down cut milling experiments were carried out to investigate the cutting phenomena resulting from the differences in the cutting direction. The experiment with a spindle speed of 10,000 min^−1^ (cutting speed of 15.7 m/min) was conducted using a machining center spindle, whereas that of 100,000 min^−1^ (cutting speed of 157 m/min) was completed using a high-speed air-turbine spindle RBX-12 (Big Daishowa Seiki Co., Higashi-Osaka, Japan). Accordingly, RBX-12 had high radial stiffness because of the rolling bearings. In the experiments, the effect of the feed rate *f* was analyzed by varying the feed speed *F* at the fixed spindle speed of 100,000 min^−1^. We conducted experiments in several times depending on the cutting conditions in order to confirm the reproducibility and reliability of the results. Meanwhile, tool wear was measured by mounting a micro end mill tool on the spindle of a machine tool with a spindle rotation control function, with the spindle precisely rotated at very low speeds. At this time, it was possible to precisely measure the profile of the cutting edge and radius reduction of the tool with the pickup of the roughness testing machine for surface roughness measurement [20]. It could be noted that most studies on tool wear [15,16,17,18,19] evaluate tool wear based on cutting edge observation; however, the method presented herein has the ability to measure details of the tool edge shape. Furthermore, the positions of the cutting edges in the axial direction of the tool were measured at five locations where the workpieces were actually machined, as this is necessary to obtain the flank wear width $V_{B}$ compared with the nose shape of the cutting edge, which was not interfered with the workpiece. The average $V_{B}$ at five points in the axial direction at 0.1 mm intervals, herein defined as the average flank wear width $\bar{V_{B}}$, was then evaluated alongside the effect of the difference in the cutting direction on the cross-sectional shape of the machined surface and the amount of residual stock removal. Cutting tests were conducted in a very delicate environment, including the condition of the machine and the chuck, and it was necessary to avoid observing the end mill on and off during each cutting test to avoid various external factors as much as possible. Because it was not possible to observe the cutting edge in detail on the machine tool, a new end mill was prepared for the cutting test at each predetermined cutting length. The average flank wear width $\bar{V_{B}}$ of the tools after each predetermined cutting length was measured. Based on the data, graphs organized by the average flank wear width $\bar{V_{B}}$ was created. The cutting length *L* machined was approximately 9 m, leaving a portion of the surface just before the workpiece every four passes. Subsequently, the accumulated amount of residual stock removal was determined by measuring the step on the machined surface of the first pass as the reference surface. This amount represents the difference between the actual and ideal machined surfaces, i.e., the machining error, including the amount of residual stock removal per pass and the amount of tool edge retreat. The cross-sectional profile of the machined surface was measured using the surface texture measuring instrument SV3100C (Mitutoyo Corp., Kanagawa, Japan).

## 3. Experimental Results and Discussion

### 3.1. Improvement of Machined Surface Quality by High-Speed Up Cut Milling

In this section, in order to investigate the effect of high-speed up cut milling, we first observed differences in tool wear with cutting length as a basic cutting phenomenon. Next, we evaluated the changes in cutting force, machined surface characteristics, and machining accuracy with the progress of tool wear.

**Figure 3:**
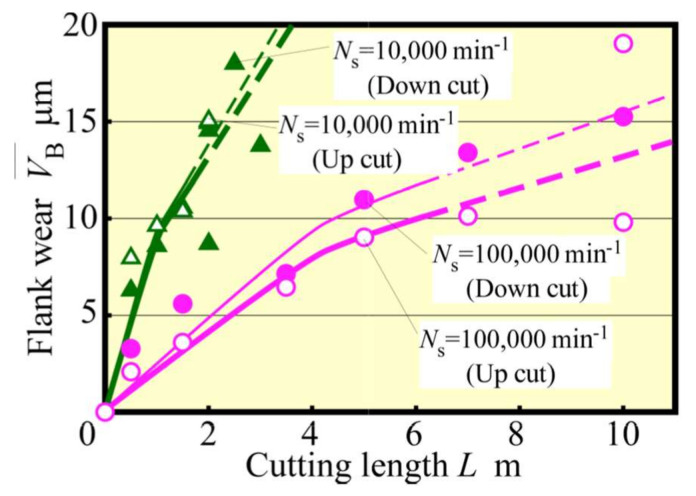
Change of flank wear with cutting length (*f* = 5 μm/tooth).

**Figure 4:**
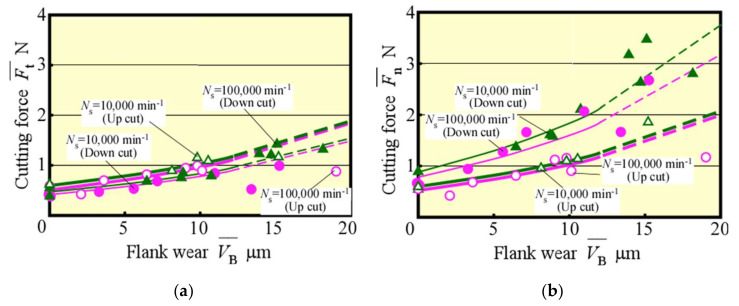
Relationship between flank wear and cutting force (*f* = 5 μm/tooth) (**a**) Normal cutting force (**b**) Tangential cutting force.

Figure 3 shows the change in $\bar{V_{B}}$ with *L*. Here, in our past research [21], it was found that the cutting phenomenon becomes unstable and the tool life was reached when the average flank wear width reaches only 12 μm in the case of the same 0.5 mm diameter end mill as the present one. Therefore, in this figure, the tendency in the area of the average flank wear width of 12 μm and more is indicated by a broken line (the same indication hereinafter). Moreover, it could be confirmed that high-speed up cut milling, i.e., at 100,000 min^−1^ spindle speed, is extremely effective in suppressing tool wear, as well as down cut milling, as indicated by an extension in the tool life by about 1.3 times, with *L* = 6–8 m, because the tool life was determined as 12 μm in tool wear for finish [20], by high-speed up cut milling. Figure 4 shows the relationship between $\bar{V_{B}}$ and the cutting forces in the steady state. The same tendency for both up and down cut milling could be observed with the change in the tangential component $\bar{F_{t}}$. By contrast, the normal component $\bar{F_{n}}$ was smaller in the up cut milling compared with that in the down cut milling operation. In particular, $\bar{F_{n}}$ increased gradually in the up cut milling before and after 12 μm of tool wear, whereas it increased rapidly after 12 μm of tool wear to cause a potentially unstable machining in the down cut milling.

**Figure 5:**
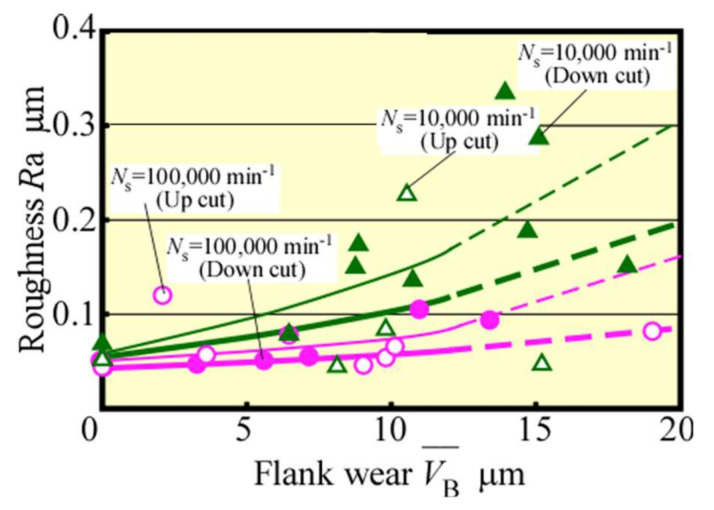
Surface roughness of the machined surface (*f* = 5 μm/tooth).

**Figure 6:**
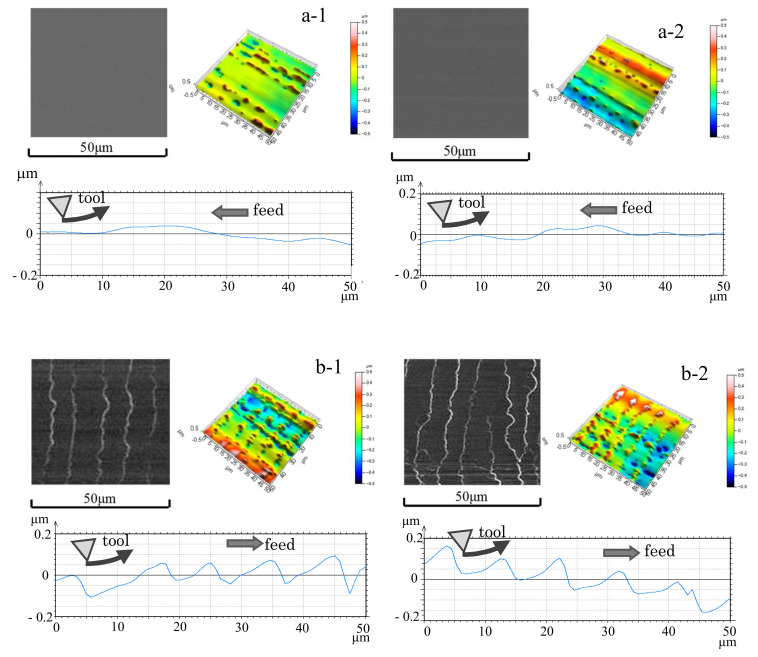
SEM images, 3D images, and profile curves of the machined surface (**a-1**) Up cut, *L* = 1.5 m (**a-2**) Up cut, *L* = 7.0 m (**b-1**) Down cut, *L* = 1.5 m (**b-2**) Down cut, *L* = 7.0 m (*N*_s_ = 100,000 min^−1^, *f* = 5 μm/tooth).

The surface roughness of the machined surface is displayed in Figure 5, which could be seen to be better in high-speed up cut milling compared with the other conditions. Figure 6 shows the SEM-observed machined surface images, along with 3D images and profile curves in the tool feed direction measured by a non-contact 3D profiler Talysurf CCI LITE (AMETEK Co., Ltd., Berwyn, PA, USA), which were a result for the *L* range 1.5–7.0 m in 100,000 min^−1^ of spindle speed. A cutting mark with an approximately 10 μm pitch corresponding to the tool feed per rotation could be observed on the machined surface in the case of down cut milling. Appropriately, the cutting mark was a material-raised shape in the cutting edge rotation direction, probably caused by burr generation in the cutting edge leaves from workpiece remains on the machined surface. In contrast, the same cutting mark was not observed on the machined surface in the up cut milling. In particular, the surface was very smooth, which could be due to material removal or as a burnishing effect by the next cutting edge. Moreover, in the case of up cut milling, the waviness component was smaller than that in the down cut milling, indicating that it can be processed more consistently in the former. Comparing the conditions of the machined surface in the 3D images, it can be said that the unevenness not only in the feed direction but also in the height direction was small and that the machined surface was good.

**Figure 7:**
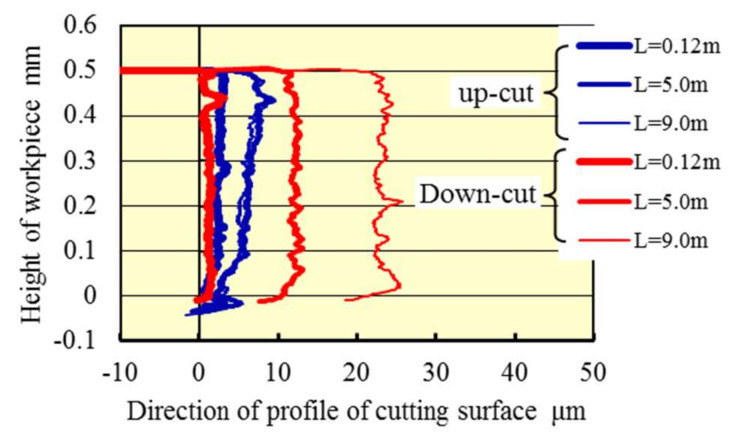
Machined surface profile (*N*_s_ = 100,000 min^−1^).

Figure 7 shows profiles of the machined surface in the spindle speed of 100,000 min^−1^, offset by each residual stock removal measured. An ideal profile of the machined surface overlaps the vertical line at zero in the horizontal line. Thus, Figure 7 indicates that the dimension error in the high-speed up cut milling was smaller than that in the down cut milling even when the cutting length became larger. The result likewise indicates that the machining accuracy in the high-speed up cut milling was better than that in the down cut milling, which probably was due to the smaller residual stock removal per path and smaller tool wear in the up cut milling. Furthermore, the dimension error became larger in response to the larger residual stock removal generated by a larger elastic deformation of the tool and the workpiece due to the large normal cutting force. Also, as explained in Figure 6, the small unevenness in the high direction in the case of up cut milling is effective in obtaining good machining accuracy. Complex tool deformation behavior of the small diameter end mill with torsional angle during machining and its effect on machining phenomena and quality will be investigated and clarified as future works.

### 3.2. Influence of Higher Feed Rate Milling

In Section 3.1., it was revealed that high-speed up cut milling can improve the machining quality. Thus, the influence of feed rate *f* was investigated to achieve more efficient machining.

**Figure 8:**
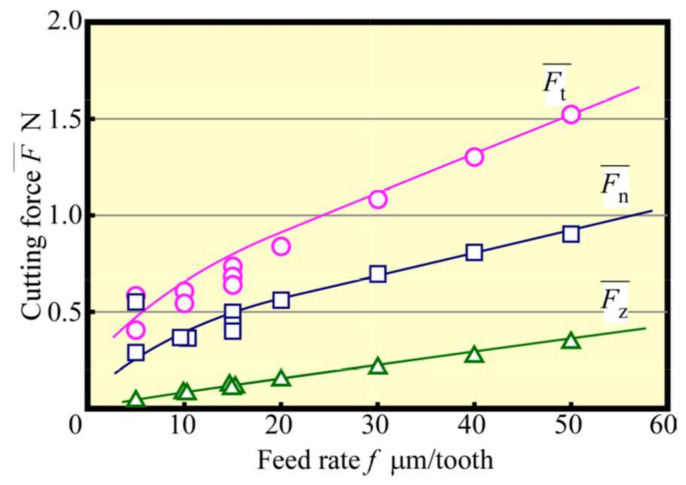
Relationship between cutting force and feed rate (Up cut, *N*_s_ = 100,000 min^−1^).

**Figure 9:**
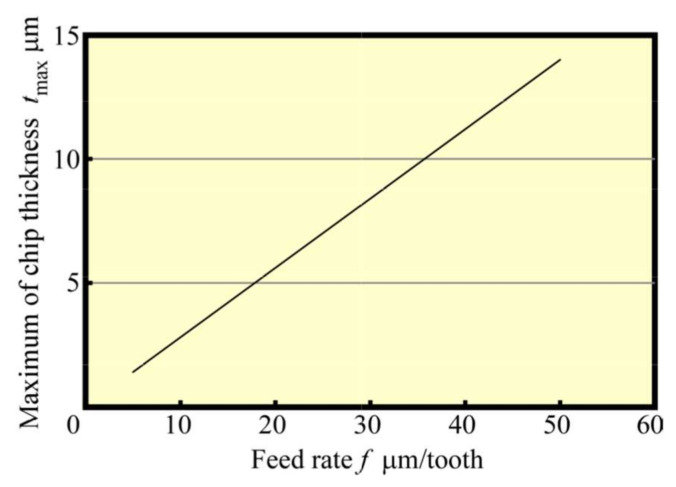
Relationship between maximum chip thickness and feed rate (*N*_s_ = 100,000 min^−1^).

**Figure 10:**
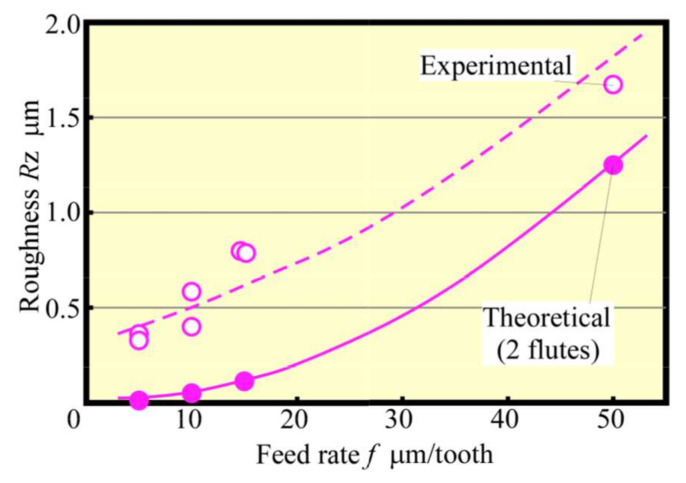
Relationship between roughness and feed rate (Up cut, *N*_s_ = 100,000 min^−1^).

Figure 8 shows that the change in the average cutting force with *f* was varied at 5–50 μm/tooth at the early stage of the cutting process. It could be seen that the average cutting force increased with the feed rate, although they were not proportional to each other, which contradicts the almost proportional relationship between the maximum geometric depth of cut (maximum chip thickness $t_{\max}$) and *f*, as depicted in Figure 9. From Figure 9, the increase rate was large when *f* was 15 μm/tooth or less; when *f* was small, the ratio of the radius of curvature of the tool edge to the depth of cut was large, resulting in poor cutting ability, whereas the average cutting force seemingly changed. Figure 10 shows the relationship between *f* and the machined surface roughness. The experimental value of machined surface roughness was approximately 0.5 μm greater than the theoretical value $Rz_{\mathrm{th}}$, expressed by Equation (1):

(1) $$Rz_{\mathrm{th}}=\frac{f^{2}}{8r}$$

where *r* is the rotation radius of the cutting edge of the tool.

**Figure 11:**
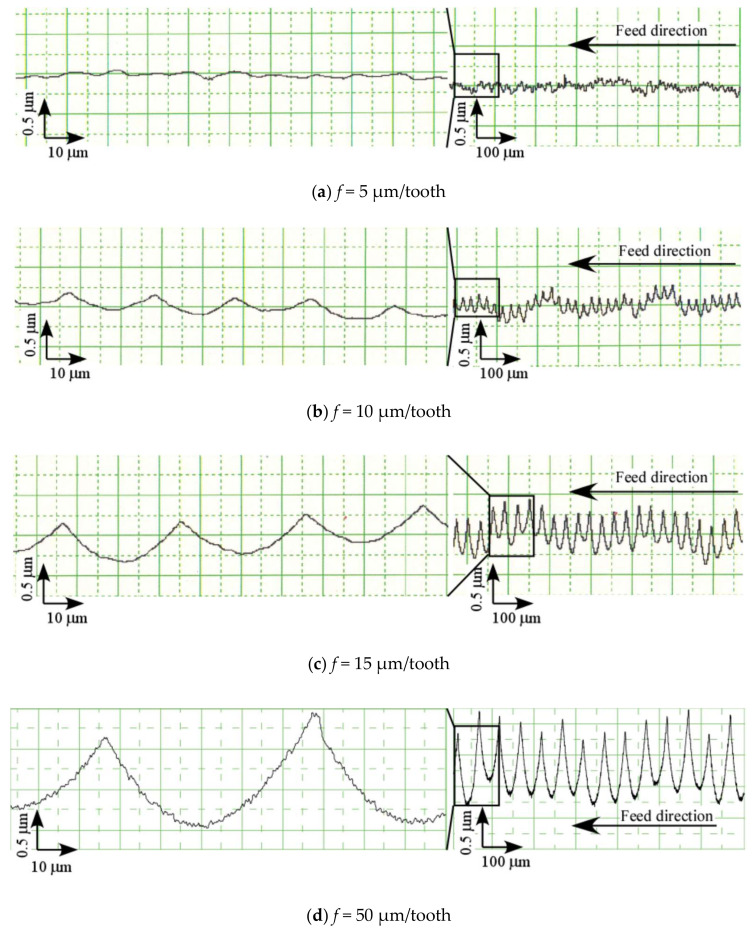
Roughness curves (Up cut, *N*_s_ = 100,000 min^−1^), (**a**) *f* = 5 µm/tooth, (**b**) *f* = 10 µm/tooth, (**c**) *f* = 15 µm/tooth, (**d**) *f* = 50 µm/tooth.

Figure 11 shows the roughness curve of the machined surface for each feed rate. The entire roughness curve shows waviness, and this seemed to have a large effect on the experimental values of surface roughness. However, zooming in the curve would display tool marks (cusps). For example, in the cutting range of one tool revolution, two tool marks remained at *f* = 50 μm/tooth, whereas only one remained at *f* = 5–15 μm/tooth. Table 2 shows the results of $Rz_{\mathrm{th}}$ for each feed rate obtained using Equation (1) and the value of *f* when the feed rate was 2*f* per tool revolution (assuming a tool mark was generated with one cutting edge). Please note that when the feed rate was small, such as *f* = 5–15 µm/tooth, a feed rate of 2*f* at the theoretical roughness $Rz_{\mathrm{th}}$ was closer to the experimental value. Although in the experiments the static runout of the tool was kept below 1 μm, the high-frequency component of the roughness curve was in sub-micrometer. The tool runout must be smaller than runout of the tool to produce a machined surface with a two-flute cutting surface. A discussion in terms of dynamic runout is necessary to elaborate on this condition. However, because it is almost practically impossible for the runout of the tool to become zero, the generation of a machined surface at a small feed rate per tooth could have suggestively been because only one cutting surface remains, even for a multi-flute tool. 

**Figure 12:**
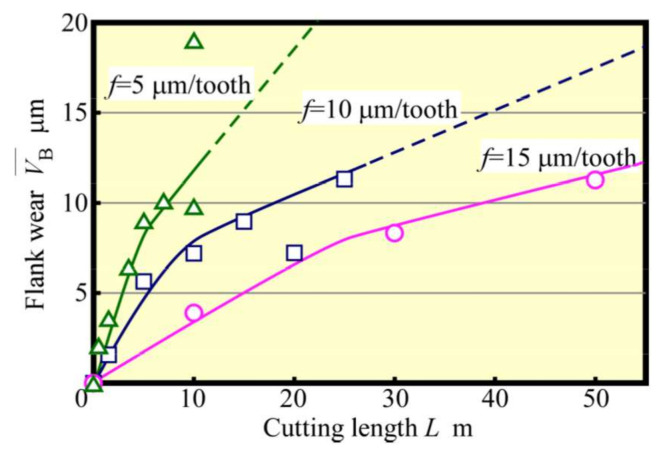
Relationship between flank wear and cutting length (Up cut, *N*_s_ = 100,000 min^−1^).

**Figure 13:**
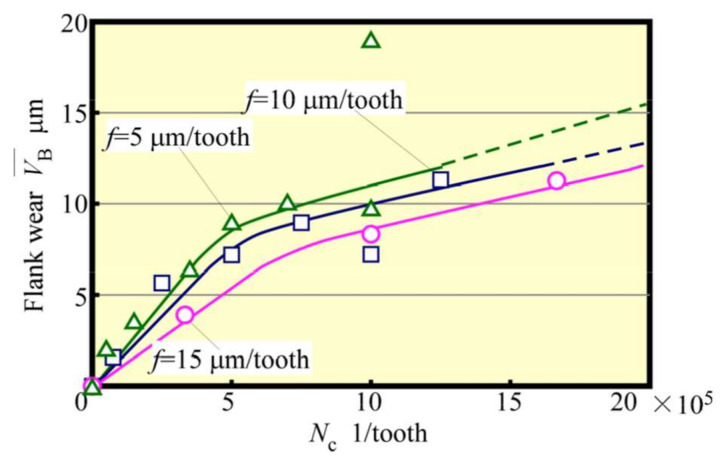
Relationship between flank wear and *N*_c_ (Up cut, *N*_s_ = 100,000 min^−1^).

**Figure 14:**
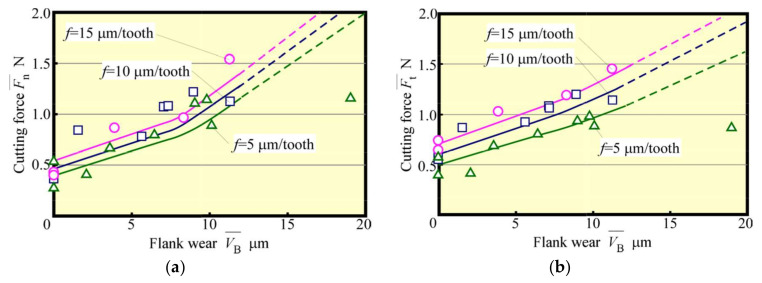
Relationship between cutting force and flank wear (Up cut, *N*_s_ = 100,000 min^−1^) (**a**) Normal cutting force (**b**) Tangential cutting force.

**Figure 15:**
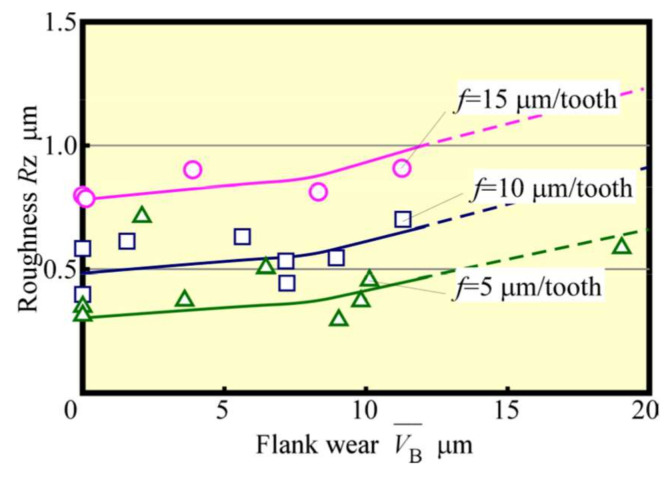
Relationship between roughness and flank wear (Up cut, *N*_s_ = 100,000 min^−1^).

Next, tool life tests were conducted for feed rates *f* = 5 μm/tooth, 10 μm/tooth, and 15 μm/tooth. The relationship between $\bar{V_{B}}$ and *L* is depicted in Figure 12. Here, the tool-life length $L_{f}$ could be seen to increase with *f*. Also, an increase in *f* tended to decrease the number of contacts $N_{c}$ between the cutting edge and the workpiece, even when the value of *L* was the same. Such an effect is considered to be significant. The relationship between $\bar{V_{B}}$ and $N_{c}$ is shown in Figure 13. When the radius of the cutting edge is relatively large compared with the amount of the cutting edge depth of cut, as in the present condition, the effect of the latter on tool wear is small. Furthermore, the effect of the number of contacts is significant unless chipping due to overload occurs. Considering the magnitude of the spring back of the workpiece, the cutter angle of the small diameter tool must be small. Therefore, it is not a good strategy to increase the impact force, as in the case of a down cut. Moreover, as depicted in Figure 13, the wear progresses more slowly when the feed rate *f* is large. It is thought that the increased feed rate *f* shortens the distance the cutting edge slides elastically over the workpiece and reduces wear due to friction. This implies that when *f* is small, the distance elastically slipping becomes longer, which creates better cutting conditions for the cutting edge to be bitten and cut without impact and without slipping. Figure 14 and Figure 15 show the relationships between the average cutting force and $\bar{V_{B}}$ and between Rz and $\bar{V_{B}}$, respectively. As in the early stage of cutting, as tool wear progressed, the feed rate became larger, and the cutting force and surface roughness values became greater. Cutting force and surface roughness are affected by tool wear, and the tendency for these values to increase with tool wear is the same regardless of the feed rate. With feed rate *f* = 15 µm/tooth, the surface roughness of the machined surface was about twice as great, or about 1 µm, as this value of *f*, even when cutting more than five times this feed rate. Therefore, it was clarified that increasing the feed rate in accordance with the required accuracy (high-speed and high-feed rate) will result in a highly efficient and long tool life machining unless chipping of the cutting edge occurs because of overload.

## 4. Conclusions

The effect of high-speed up cut milling on micro end milling for hardened die steel with a 0.5 mm diameter end mill tool was investigated herein with the following results:

- In high-speed up cut milling, with say 157 m/min (100,000 min^−1^) of cutting speed, the progress of tool wear can be suppressed. This results in an increase of approximately 30% of tool life compared with that in down cut milling.
- High-speed up cut milling improves not only the surface roughness but also the dimensional accuracy.
- The machined surface in high-speed up cut milling is smooth with non-observable cutting marks.
- At a small feed rate, undulation occurs on the machined surface, which is generated by the single-flute cutting surface.
- High-efficiency and long tool life machining can be achieved by high-speed up cut milling and high-feed cutting, unless chipping due to overload occurs.

The effect of deformation due to low stiffness of a small diameter end mill on cutting phenomena will be investigated as a future research issue.

## Figures and Tables

**Table 1:** Cutting conditions.

**Workpiece**	JIS: SKD61 53HRC
**Spindle Speed *N*_s_, min^−1^** **(Cutting Speed *V*_c_, m/min)**	10,000, 100,000 (15.7), (157)
**Feed Rate *f*, μm/tooth**	5–50
**Radial Depth of cut *R*_d_, μm**	10
**Axial Depth of cut *A*_d_, mm**	0.5
**Cutting Length, *L* m**	0–10
**Cutting Direction**	Up Cut, Down Cut
**Tool Run Out, μm**	<1
**Coolant**	Dry Air

**Table 2:** Theoretical roughness.

Feed Rate *f* µm/Tooth	Theoretical Roughness (2 Flutes) µm	Theoretical Roughness (1 Flute) µm
**5**	0.013	0.050
**10**	0.050	0.200
**15**	0.113	0.450
**20**	0.200	0.800
**30**	0.450	1.800
**50**	1.250	5.000
